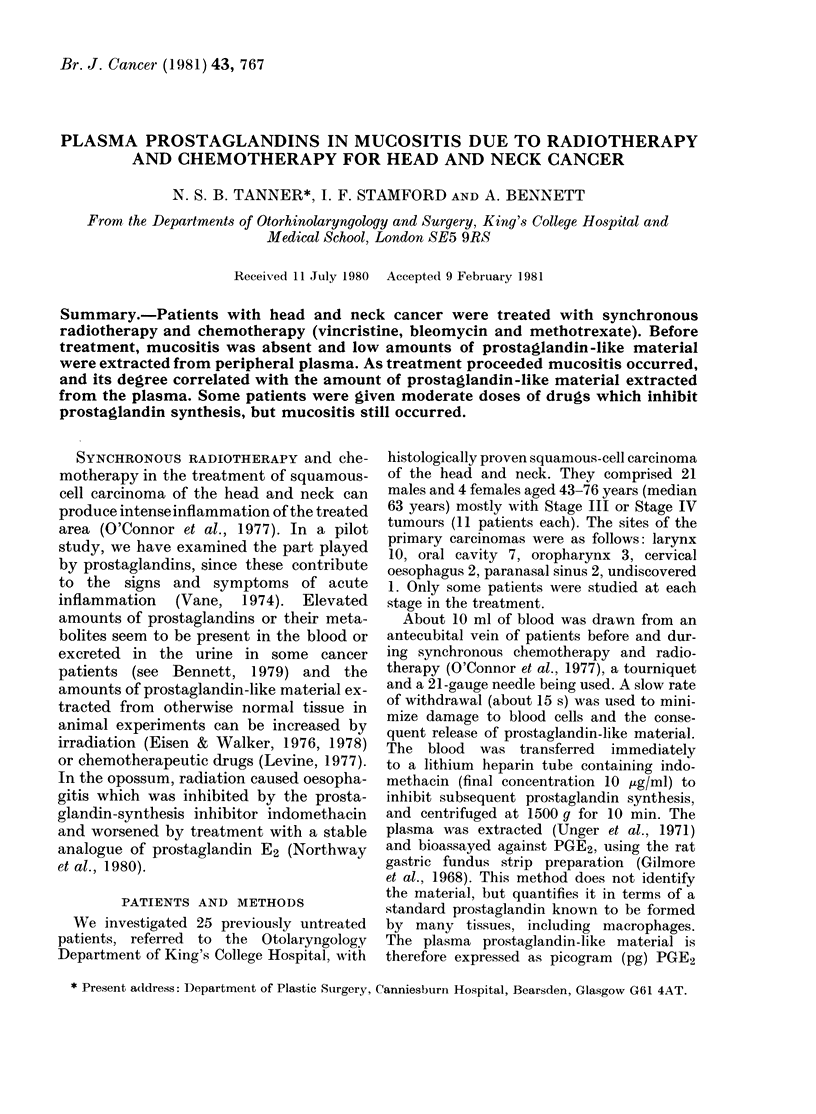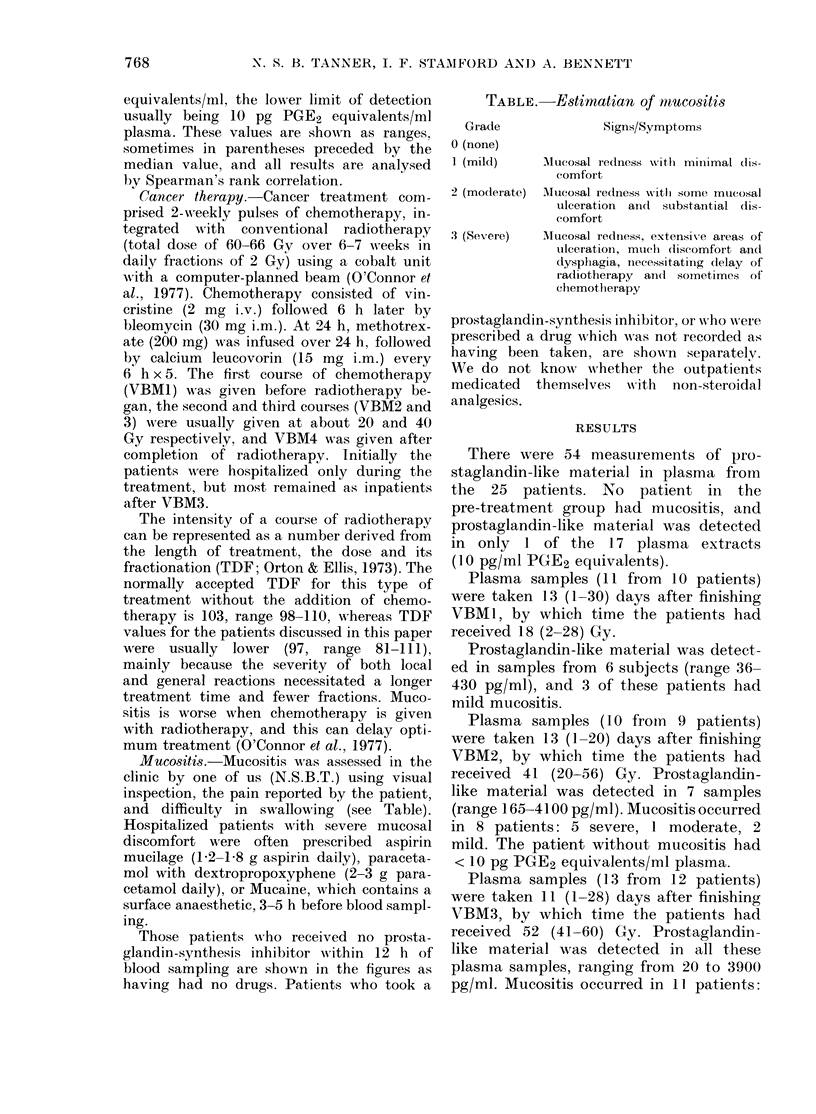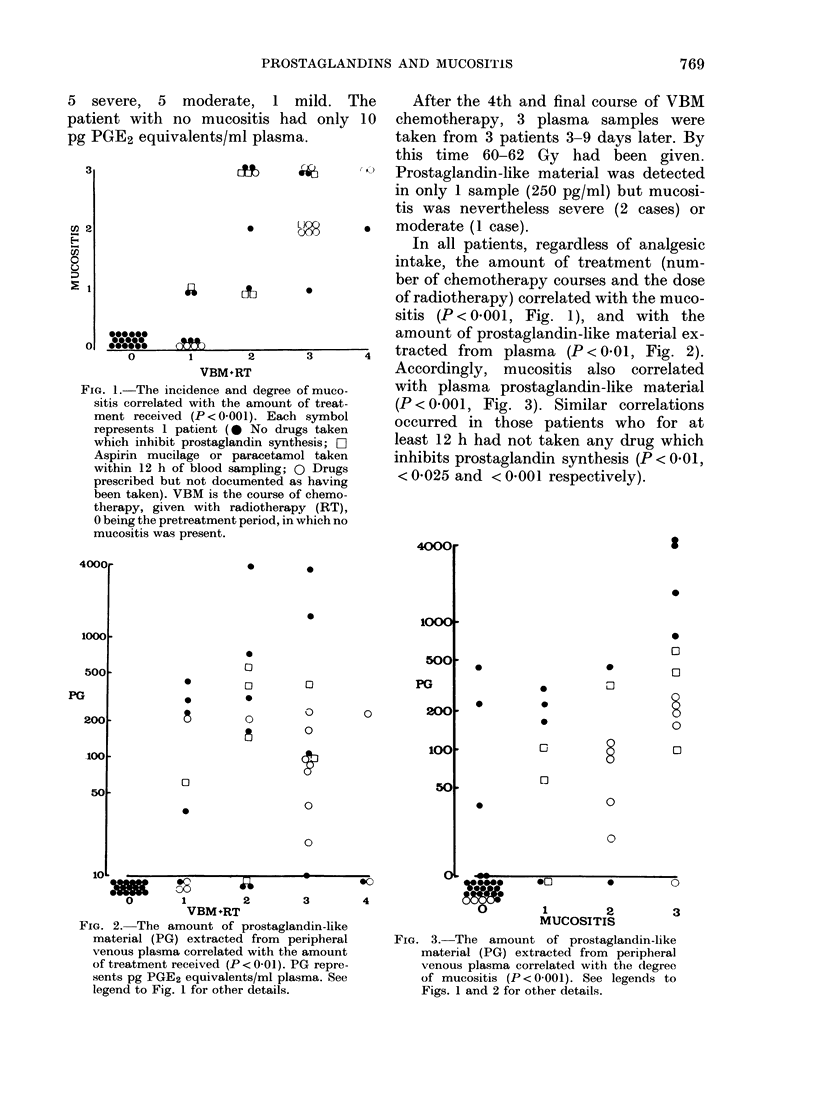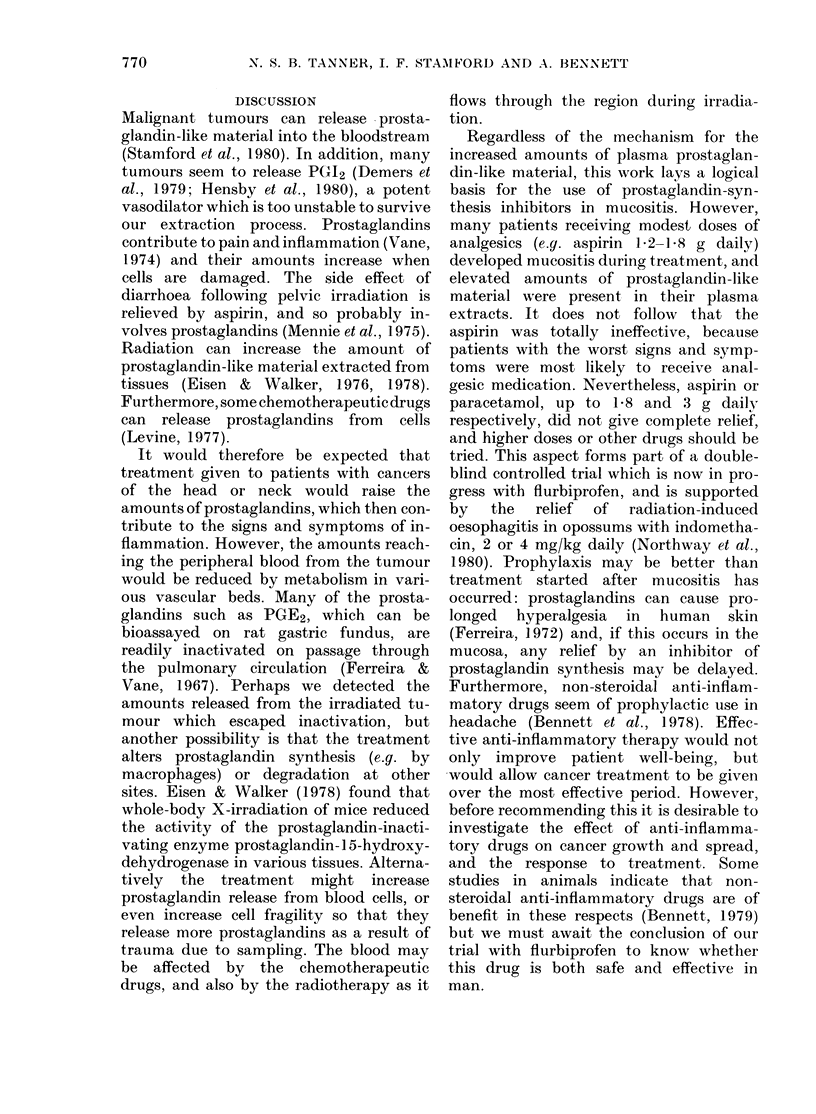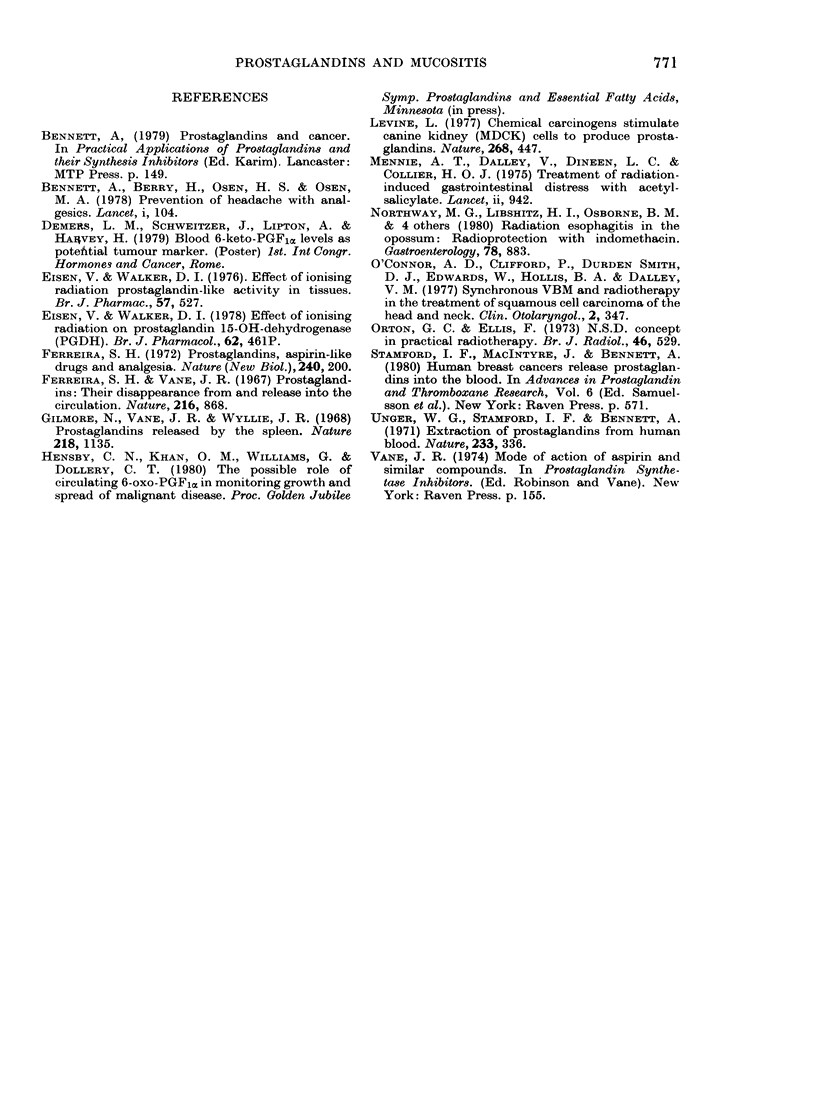# Plasma prostaglandins in mucositis due to radiotherapy and chemotherapy for head and neck cancer.

**DOI:** 10.1038/bjc.1981.114

**Published:** 1981-06

**Authors:** N. S. Tanner, I. F. Stamford, A. Bennett

## Abstract

Patients with head and neck cancer were treated with synchronous radiotherapy and chemotherapy (vincristine, bleomycin and methotrexate). Before treatment, mucositis was absent and low amounts of prostaglandin-like material were extracted from peripheral plasma. As treatment proceeded mucositis occurred, and its degree correlated with the amount of prostaglandin-like material extracted from the plasma. Some patients were given moderate doses of drugs which inhibit prostaglandin synthesis, but mucositis still occurred.


*Br. J. Cancer* (1981) **43**, 767

### PLASMA PROSTAGLANDINS IN MUCOSITIS DUE TO RADIOTHERAPY AND CHEMOTHERAPY FOR HEAD AND NECK CANCER

N. S. B. TANNER*, I. F. STAMFORD AND A. BENNETT

*From the Departments of Otorhinolaryngology and Surgery, King's College Hospital and Medical School, London SE5 9RS*

Received 11 July 1980 Accepted 9 February 1981

**Summary.—Patients with head and neck cancer were treated with synchronous radiotherapy and chemotherapy (vincristine, bleomycin and methotrexate). Before treatment, mucositis was absent and low amounts of prostaglandin-like material were extracted from peripheral plasma. As treatment proceeded mucositis occurred, and its degree correlated with the amount of prostaglandin-like material extracted from the plasma. Some patients were given moderate doses of drugs which inhibit prostaglandin synthesis, but mucositis still occurred.**

SYNCHRONOUS RADIOTHERAPY and chemotherapy in the treatment of squamous-cell carcinoma of the head and neck can produce intense inflammation of the treated area (O'Connor *et al.*, 1977). In a pilot study, we have examined the part played by prostaglandins, since these contribute to the signs and symptoms of acute inflammation (Vane, 1974). Elevated amounts of prostaglandins or their metabolites seem to be present in the blood or excreted in the urine in some cancer patients (see Bennett, 1979) and the amounts of prostaglandin-like material extracted from otherwise normal tissue in animal experiments can be increased by irradiation (Eisen & Walker, 1976, 1978) or chemotherapeutic drugs (Levine, 1977). In the opossum, radiation caused oesophagitis which was inhibited by the prostaglandin-synthesis inhibitor indomethacin and worsened by treatment with a stable analogue of prostaglandin $E_2$ (Northway *et al.*, 1980).

### PATIENTS AND METHODS

We investigated 25 previously untreated patients, referred to the Otolaryngology Department of King's College Hospital, with histologically proven squamous-cell carcinoma of the head and neck. They comprised 21 males and 4 females aged 43–76 years (median 63 years) mostly with Stage III or Stage IV tumours (11 patients each). The sites of the primary carcinomas were as follows: larynx 10, oral cavity 7, oropharynx 3, cervical oesophagus 2, paranasal sinus 2, undiscovered 1. Only some patients were studied at each stage in the treatment.

About 10 ml of blood was drawn from an antecubital vein of patients before and during synchronous chemotherapy and radiotherapy (O'Connor *et al.*, 1977), a tourniquet and a 21-gauge needle being used. A slow rate of withdrawal (about 15 s) was used to minimize damage to blood cells and the consequent release of prostaglandin-like material. The blood was transferred immediately to a lithium heparin tube containing indomethacin (final concentration 10 $\mu$g/ml) to inhibit subsequent prostaglandin synthesis, and centrifuged at 1500 *g* for 10 min. The plasma was extracted (Unger *et al.*, 1971) and bioassayed against $PGE_2$, using the rat gastric fundus strip preparation (Gilmore *et al.*, 1968). This method does not identify the material, but quantifies it in terms of a standard prostaglandin known to be formed by many tissues, including macrophages. The plasma prostaglandin-like material is therefore expressed as picogram (pg) $PGE_2$

* Present address: Department of Plastic Surgery, Canniesburn Hospital, Bearsden, Glasgow G61 4AT.

equivalents/ml, the lower limit of detection usually being 10 pg $PGE_2$ equivalents/ml plasma. These values are shown as ranges, sometimes in parentheses preceded by the median value, and all results are analysed by Spearman's rank correlation.

*Cancer therapy.*—Cancer treatment comprised 2-weekly pulses of chemotherapy, integrated with conventional radiotherapy (total dose of 60–66 Gy over 6–7 weeks in daily fractions of 2 Gy) using a cobalt unit with a computer-planned beam (O'Connor *et al.*, 1977). Chemotherapy consisted of vincristine (2 mg i.v.) followed 6 h later by bleomycin (30 mg i.m.). At 24 h, methotrexate (200 mg) was infused over 24 h, followed by calcium leucovorin (15 mg i.m.) every 6 h × 5. The first course of chemotherapy (VBM1) was given before radiotherapy began, the second and third courses (VBM2 and 3) were usually given at about 20 and 40 Gy respectively, and VBM4 was given after completion of radiotherapy. Initially the patients were hospitalized only during the treatment, but most remained as inpatients after VBM3.

The intensity of a course of radiotherapy can be represented as a number derived from the length of treatment, the dose and its fractionation (TDF; Orton & Ellis, 1973). The normally accepted TDF for this type of treatment without the addition of chemotherapy is 103, range 98–110, whereas TDF values for the patients discussed in this paper were usually lower (97, range 81–111), mainly because the severity of both local and general reactions necessitated a longer treatment time and fewer fractions. Mucositis is worse when chemotherapy is given with radiotherapy, and this can delay optimum treatment (O'Connor *et al.*, 1977).

*Mucositis.*—Mucositis was assessed in the clinic by one of us (N.S.B.T.) using visual inspection, the pain reported by the patient, and difficulty in swallowing (see Table). Hospitalized patients with severe mucosal discomfort were often prescribed aspirin mucilage (1·2–1·8 g aspirin daily), paracetamol with dextropropoxyphene (2–3 g paracetamol daily), or Mucaine, which contains a surface anaesthetic, 3–5 h before blood sampling.

Those patients who received no prostaglandin-synthesis inhibitor within 12 h of blood sampling are shown in the figures as having had no drugs. Patients who took a prostaglandin-synthesis inhibitor, or who were prescribed a drug which was not recorded as having been taken, are shown separately. We do not know whether the outpatients medicated themselves with non-steroidal analgesics.

TABLE.—*Estimatian of mucositis*

| Grade | Signs/Symptoms |
|---|---|
| 0 (none) | |
| 1 (mild) | Mucosal redness with minimal discomfort |
| 2 (moderate) | Mucosal redness with some mucosal ulceration and substantial discomfort |
| 3 (Severe) | Mucosal redness, extensive areas of ulceration, much discomfort and dysphagia, necessitating delay of radiotherapy and sometimes of chemotherapy |

### RESULTS

There were 54 measurements of prostaglandin-like material in plasma from the 25 patients. No patient in the pre-treatment group had mucositis, and prostaglandin-like material was detected in only 1 of the 17 plasma extracts (10 pg/ml $PGE_2$ equivalents).

Plasma samples (11 from 10 patients) were taken 13 (1–30) days after finishing VBM1, by which time the patients had received 18 (2–28) Gy.

Prostaglandin-like material was detected in samples from 6 subjects (range 36–430 pg/ml), and 3 of these patients had mild mucositis.

Plasma samples (10 from 9 patients) were taken 13 (1–20) days after finishing VBM2, by which time the patients had received 41 (20–56) Gy. Prostaglandin-like material was detected in 7 samples (range 165–4100 pg/ml). Mucositis occurred in 8 patients: 5 severe, 1 moderate, 2 mild. The patient without mucositis had $< 10$ pg $PGE_2$ equivalents/ml plasma.

Plasma samples (13 from 12 patients) were taken 11 (1–28) days after finishing VBM3, by which time the patients had received 52 (41–60) Gy. Prostaglandin-like material was detected in all these plasma samples, ranging from 20 to 3900 pg/ml. Mucositis occurred in 11 patients:

5 severe, 5 moderate, 1 mild. The patient with no mucositis had only 10 pg $PGE_2$ equivalents/ml plasma.

FIG. 1.—The incidence and degree of mucositis correlated with the amount of treatment received ($P < 0{\cdot}001$). Each symbol represents 1 patient (● No drugs taken which inhibit prostaglandin synthesis; □ Aspirin mucilage or paracetamol taken within 12 h of blood sampling; ○ Drugs prescribed but not documented as having been taken). VBM is the course of chemotherapy, given with radiotherapy (RT), 0 being the pretreatment period, in which no mucositis was present.

FIG. 2.—The amount of prostaglandin-like material (PG) extracted from peripheral venous plasma correlated with the amount of treatment received ($P < 0{\cdot}01$). PG represents pg $PGE_2$ equivalents/ml plasma. See legend to Fig. 1 for other details.

After the 4th and final course of VBM chemotherapy, 3 plasma samples were taken from 3 patients 3–9 days later. By this time 60–62 Gy had been given. Prostaglandin-like material was detected in only 1 sample (250 pg/ml) but mucositis was nevertheless severe (2 cases) or moderate (1 case).

In all patients, regardless of analgesic intake, the amount of treatment (number of chemotherapy courses and the dose of radiotherapy) correlated with the mucositis ($P < 0{\cdot}001$, Fig. 1), and with the amount of prostaglandin-like material extracted from plasma ($P < 0{\cdot}01$, Fig. 2). Accordingly, mucositis also correlated with plasma prostaglandin-like material ($P < 0{\cdot}001$, Fig. 3). Similar correlations occurred in those patients who for at least 12 h had not taken any drug which inhibits prostaglandin synthesis ($P < 0{\cdot}01$, $< 0{\cdot}025$ and $< 0{\cdot}001$ respectively).

FIG. 3.—The amount of prostaglandin-like material (PG) extracted from peripheral venous plasma correlated with the degree of mucositis ($P < 0{\cdot}001$). See legends to Figs. 1 and 2 for other details.

### DISCUSSION

Malignant tumours can release prostaglandin-like material into the bloodstream (Stamford *et al.*, 1980). In addition, many tumours seem to release $PGI_2$ (Demers *et al.*, 1979; Hensby *et al.*, 1980), a potent vasodilator which is too unstable to survive our extraction process. Prostaglandins contribute to pain and inflammation (Vane, 1974) and their amounts increase when cells are damaged. The side effect of diarrhoea following pelvic irradiation is relieved by aspirin, and so probably involves prostaglandins (Mennie *et al.*, 1975). Radiation can increase the amount of prostaglandin-like material extracted from tissues (Eisen & Walker, 1976, 1978). Furthermore, some chemotherapeutic drugs can release prostaglandins from cells (Levine, 1977).

It would therefore be expected that treatment given to patients with cancers of the head or neck would raise the amounts of prostaglandins, which then contribute to the signs and symptoms of inflammation. However, the amounts reaching the peripheral blood from the tumour would be reduced by metabolism in various vascular beds. Many of the prostaglandins such as $PGE_2$, which can be bioassayed on rat gastric fundus, are readily inactivated on passage through the pulmonary circulation (Ferreira & Vane, 1967). Perhaps we detected the amounts released from the irradiated tumour which escaped inactivation, but another possibility is that the treatment alters prostaglandin synthesis (*e.g.* by macrophages) or degradation at other sites. Eisen & Walker (1978) found that whole-body X-irradiation of mice reduced the activity of the prostaglandin-inactivating enzyme prostaglandin-15-hydroxydehydrogenase in various tissues. Alternatively the treatment might increase prostaglandin release from blood cells, or even increase cell fragility so that they release more prostaglandins as a result of trauma due to sampling. The blood may be affected by the chemotherapeutic drugs, and also by the radiotherapy as it flows through the region during irradiation.

Regardless of the mechanism for the increased amounts of plasma prostaglandin-like material, this work lays a logical basis for the use of prostaglandin-synthesis inhibitors in mucositis. However, many patients receiving modest doses of analgesics (*e.g.* aspirin 1·2–1·8 g daily) developed mucositis during treatment, and elevated amounts of prostaglandin-like material were present in their plasma extracts. It does not follow that the aspirin was totally ineffective, because patients with the worst signs and symptoms were most likely to receive analgesic medication. Nevertheless, aspirin or paracetamol, up to 1·8 and 3 g daily respectively, did not give complete relief, and higher doses or other drugs should be tried. This aspect forms part of a double-blind controlled trial which is now in progress with flurbiprofen, and is supported by the relief of radiation-induced oesophagitis in opossums with indomethacin, 2 or 4 mg/kg daily (Northway *et al.*, 1980). Prophylaxis may be better than treatment started after mucositis has occurred: prostaglandins can cause prolonged hyperalgesia in human skin (Ferreira, 1972) and, if this occurs in the mucosa, any relief by an inhibitor of prostaglandin synthesis may be delayed. Furthermore, non-steroidal anti-inflammatory drugs seem of prophylactic use in headache (Bennett *et al.*, 1978). Effective anti-inflammatory therapy would not only improve patient well-being, but would allow cancer treatment to be given over the most effective period. However, before recommending this it is desirable to investigate the effect of anti-inflammatory drugs on cancer growth and spread, and the response to treatment. Some studies in animals indicate that non-steroidal anti-inflammatory drugs are of benefit in these respects (Bennett, 1979) but we must await the conclusion of our trial with flurbiprofen to know whether this drug is both safe and effective in man.

### REFERENCES

BENNETT, A, (1979) Prostaglandins and cancer. In *Practical Applications of Prostaglandins and their Synthesis Inhibitors* (Ed. Karim). Lancaster: MTP Press. p. 149.

BENNETT, A., BERRY, H., OSEN, H. S. & OSEN, M. A. (1978) Prevention of headache with analgesics. *Lancet*, i, 104.

DEMERS, L. M., SCHWEITZER, J., LIPTON, A. & HARVEY, H. (1979) Blood 6-keto-$PGF_{1\alpha}$ levels as potential tumour marker. (Poster) *1st. Int Congr. Hormones and Cancer, Rome.*

EISEN, V. & WALKER, D. I. (1976). Effect of ionising radiation prostaglandin-like activity in tissues. *Br. J. Pharmac.*, **57**, 527.

EISEN, V. & WALKER, D. I. (1978) Effect of ionising radiation on prostaglandin 15-OH-dehydrogenase (PGDH). *Br. J. Pharmacol.*, **62**, 461P.

FERREIRA, S. H. (1972) Prostaglandins, aspirin-like drugs and analgesia. *Nature* (*New Biol.*), **240**, 200.

FERREIRA, S. H. & VANE, J. R. (1967) Prostaglandins: Their disappearance from and release into the circulation. *Nature*, **216**, 868.

GILMORE, N., VANE, J. R. & WYLLIE, J. R. (1968) Prostaglandins released by the spleen. *Nature* **218**, 1135.

HENSBY, C. N., KHAN, O. M., WILLIAMS, G. & DOLLERY, C. T. (1980) The possible role of circulating 6-oxo-$PGF_{1\alpha}$ in monitoring growth and spread of malignant disease. *Proc. Golden Jubilee Symp. Prostaglandins and Essential Fatty Acids, Minnesota* (in press).

LEVINE, L. (1977) Chemical carcinogens stimulate canine kidney (MDCK) cells to produce prostaglandins. *Nature*, **268**, 447.

MENNIE, A. T., DALLEY, V., DINEEN, L. C. & COLLIER, H. O. J. (1975) Treatment of radiation-induced gastrointestinal distress with acetylsalicylate. *Lancet*, ii, 942.

NORTHWAY, M. G., LIBSHITZ, H. I., OSBORNE, B. M. & 4 others (1980) Radiation esophagitis in the opossum: Radioprotection with indomethacin. *Gastroenterology*, **78**, 883.

O'CONNOR, A. D., CLIFFORD, P., DURDEN SMITH, D. J., EDWARDS, W., HOLLIS, B. A. & DALLEY, V. M. (1977) Synchronous VBM and radiotherapy in the treatment of squamous cell carcinoma of the head and neck. *Clin. Otolaryngol.*, **2**, 347.

ORTON, G. C. & ELLIS, F. (1973) N.S.D. concept in practical radiotherapy. *Br. J. Radiol.*, **46**, 529.

STAMFORD, I. F., MACINTYRE, J. & BENNETT, A. (1980) Human breast cancers release prostaglandins into the blood. In *Advances in Prostaglandin and Thromboxane Research*, Vol. 6 (Ed. Samuelsson *et al.*). New York: Raven Press. p. 571.

UNGER, W. G., STAMFORD, I. F. & BENNETT, A. (1971) Extraction of prostaglandins from human blood. *Nature*, **233**, 336.

VANE, J. R. (1974) Mode of action of aspirin and similar compounds. In *Prostaglandin Synthetase Inhibitors*. (Ed. Robinson and Vane). New York: Raven Press. p. 155.